# Investigating the Relationship between Chronic Liver Cirrhosis and Parkinsonism: A Comparative Analysis and a Suggested Diagnostic Scheme

**DOI:** 10.3390/clinpract14040110

**Published:** 2024-07-11

**Authors:** Tal Sigawi, Omer Hamtzany, Noa Hurvitz, Yuval Ishay, Roy Dayan, David Arkadir, Yaron Ilan

**Affiliations:** 1 Department of Medicine, Hadassah Medical Center, Faculty of Medicine, Hebrew University, Jerusalem 9112001, Israel; sigaw@hadassah.org.il (T.S.); omerh@hadassah.org.il (O.H.); noa.hurvitz@mail.huji.ac.il (N.H.); yuvali01@hadassah.org.il (Y.I.); 2 Department of Neurology, Hadassah Medical Center, Faculty of Medicine, Hebrew University, Jerusalem 9112001, Israel; royd@hadassah.org.il (R.D.); arkadir@hadassah.org.il (D.A.)

**Keywords:** cirrhosis, liver failure, Parkinson’s, Parkinsonism, basal ganglia

## Abstract

**Aim:** Neurological manifestations are common in patients with chronic liver diseases. This study aimed to depict the association between liver cirrhosis and Parkinson’s disease (PD) and propose a clinically relevant diagnostic scheme. **Methods**: We examined patients’ medical records with PD and chronic liver impairment secondary to cirrhosis or liver metastases for temporal correlations between liver insult and Parkinsonian signs. **Results:** Thirty-five individuals with PD and chronic liver impairment were included due to either cirrhosis or liver metastases. In all 22 patients with PD and liver metastases, the diagnosis of PD preceded the diagnosis of cancer. Conversely, patients with cirrhosis were often diagnosed with liver impairment before diagnosing PD. Age at diagnosis did not account for this difference. **Conclusions:** This study reinforces the potential clinical association between cirrhosis and PD. We also provide a diagnostic scheme that may guide therapeutic interventions and prognostic assessments.

## 1. Introduction

Acute liver failure is recognized for its potential to elicit a spectrum of neurological manifestations, including Parkinsonism [1,2,3]. Additionally, decompensated chronic liver failure can lead to neurological symptoms [4].

The intricate interplay between liver and neurological dysfunction has been widely recognized in the medical literature. Despite this, the potential relationship between chronic liver cirrhosis and Parkinson’s disease (PD) remains an area that has not been extensively explored. Empirical evidence to elucidate the association between the two conditions is limited, leaving this particular relationship relatively uncharted territory in medical research.

This study aims to investigate the connection between chronic liver cirrhosis and PD, potentially paving the way for enhanced understanding, diagnosis, and management of these complex medical conditions.

## 2. Methods

We screened the electronic database of Hadassah Medical Center (Jerusalem, Israel) for records of patients diagnosed with hepatic impairment and PD. The ICD-9 codes used for the search in the electronic files included the combination of ‘Parkinson’s 332.0’ with ‘Cirrhosis 571.5’ or ‘Elevated liver function tests either 790.6.02 or 790.6.01’. We excluded individuals with a transient increment of their liver enzymes due to transient hepatic injuries from our analysis. 

To test the connections between chronic liver failure and Parkinsonism, we performed a comparative analysis of time intervals between the diagnosis of liver insult and Parkinsonism in two distinct populations: individuals with liver cirrhosis and those with liver metastases, serving as a control group. 

The IRB committee of the Hadassah Medical Center in Jerusalem approved the study. Study No. (0081-23-HMO); no informed consent was required.

## 3. Results

We traced 54 individuals with PD and impaired hepatic function. Nineteen individuals had a transient hepatic impairment and were excluded from our study. Based on the available etiology of hepatic impairment, we divided the patients into two groups:

a. PD with non-metastatic cirrhotic liver disease: 13/35 (37.1%) of the patients were diagnosed with liver cirrhosis. Liver cirrhosis that was attributed to viral hepatitis was the most common cause (6/13, 46%, including hepatitis C in 3/13, hepatitis B in 2/13, and combined hepatitis C and B infection in 1/13), followed by drug-induced liver injury (2/13, 16.7%), nonalcoholic steatohepatitis (NASH, 2/13, 15.4%, one of them also with evidence of autoimmune hepatitis in biopsy), and primary biliary cirrhosis (PBC, 1/13, 7.7%). In 2/13 patients (15.4%), the etiology was not found. 

The median patient’s age at the PD diagnosis was 70 years (range 58–84; data were available for 11/13 individuals). The median age at diagnosis of cirrhosis was 71 years (range 56–85 years; data were available for 9/13 individuals).

b. PD with non-cirrhotic, metastatic hepatic impairment: 22/35 (62.9%) of the patients with PD and chronic liver impairment had non-cirrhotic hepatic impairment secondary to metastatic disease. The most common origin of metastases was the gastrointestinal tract (12/22, 54.6%), followed by the pancreas (3/22, 13.6%), lung (2/22, 9.1%), and prostate cancer (2/22, 9.1%). The remaining three patients had metastases of unknown origin.

The median patient’s age at the PD diagnosis was 71 years (56–86 years; data were available for 17/22 individuals). The median age at diagnosis of liver metastatic disease was 74.5 years (range 64–89 years; data were available for 22/22 individuals).

### Temporal Relations between PD and Liver Disease

In all 22 patients with PD and metastatic hepatic impairment, PD diagnosis preceded liver neoplasia diagnosis. Data regarding the exact time interval between diagnosis of PD and liver metastases were available for 17/22 patients. They revealed a median interval of 3 years (PD preceded cancer diagnosis in a range of 1–10 years). 

Data regarding the temporal association between the diagnosis of PD and non-metastatic liver cirrhosis were available for 9/13 patients. In four of the patients (44.4%), PD diagnosis followed the diagnosis of cirrhosis. PD was diagnosed earlier in the other four patients (44.4%). In one patient, both diagnoses were given in the same year. Overall, the average interval between diagnoses reveals that PD preceded cirrhosis in 1 year (median 0 years, range of 11 years before to 8 years after cirrhosis diagnosis).

The time interval between diagnoses was statistically significant (two-tailed Mann–Whitney U test, *p* value = 0.019). This difference was not explained by the age of PD diagnosis (two-tailed Mann–Whitney U test, *p* value = 0.77) or the age of liver disease (two-tailed Mann–Whitney U test, *p* value = 0.11). 

## 4. Discussion

The study results indicate a difference in the occurrence of PD diagnosis between patients with metastatic liver disease and those with cirrhosis. Specifically, individuals with metastatic liver disease commonly exhibit Parkinsonian signs before receiving a cancer diagnosis. Conversely, patients with cirrhosis may display these signs before or after being diagnosed with liver failure. Importantly, this disparity is not linked to variations in the age at which both conditions are diagnosed.

These findings imply that liver disease in the context of cirrhosis may contribute to the development of PD, whereas such causal relationships are less likely in patients with metastatic disease. We hypothesize that cirrhosis and metastases-associated liver failure possess distinct biochemical and functional characteristics that may account for this difference.

### 4.1. The Potential Associations between Chronic Liver Disease and Parkinsonism 

Patients with liver cirrhosis are at increased risk for a broad spectrum of neurologic complications. These can be divided into direct effects of cirrhosis on the nervous system, including hepatic encephalopathy, acquired hepatocerebral degeneration, cirrhotic myelopathy, and neurologic complications related to specific etiologies of liver disease, such as chronic alcohol use, Wilson’s disease, and HCV-related CNS complications [4]. 

The association between basal ganglia and liver disease was first described as part of Wilson’s disease as familial hepatolenticular degeneration [5]. Soon after, a chronic neurological involvement in cases of advanced cirrhosis, unrelated to Wilson’s disease, was described. Later, this involvement was named acquired chronic hepato-cerebral degeneration (ACHD) or acquired hepatolenticular degeneration (AHD) [6]. AHD is clinically distinct from hepatic encephalopathy. It is a chronic, persistent syndrome predominated by symptoms related to basal ganglia dysfunction. Altered MRI signals from the basal ganglia have been consistently reported in AHD.

1. Acquired hepatolenticular degeneration, also known as “Parkinsonism in cirrhosis”

Acquired hepatolenticular degeneration (AHD) presents a variety of neurological syndromes consisting of various movement disorders and cognitive impairment in advanced liver cirrhosis. The prevalence of AHD and movement disorders varies widely and affects up to 50% of patients diagnosed with cirrhosis [7]. Parkinsonism is the most common movement disorder; its prevalence varies between 0.8 to 21% in most studies but affects up to 50% of the patients with AHD [8,9]. In a study that showed a relatively low incidence (3.5%) of Parkinsonism in cirrhosis during a 7-year follow-up, the low incidence was twice as high as in the population without cirrhosis [10]. Other neurological symptoms include progressive ataxia, dystonia, choreoathetosis, and spastic paraparesis. 

The pathophysiology is not entirely clear, but there are clues that manganese accumulates in the basal ganglia and damages dopaminergic motor pathways owing to the presence of portosystemic shunts. This finding is supported by neuroimaging modalities and high plasma manganese levels close to occupational toxicity levels [9,11]. 

AHD, also known as “Parkinsonism in cirrhosis”, is characterized by extrapyramidal symptoms, including hypokinesia, dystonia, and rigidity. It is a rapidly progressive disease and may be independent of the severity of cognitive dysfunction. The clinical syndrome is similar in its characteristics to PD, including bradykinesia, postural instability, action, postural tremor, and gait abnormality [8,9]. Unlike classical PD, cirrhosis-related Parkinsonism presents with early gait and balance dysfunction, the relative absence of resting tremor, mild cognitive impairment at the time of presentation, elevated serum manganese levels, and little or no response to levodopa therapy [4]. The clinical deterioration is rapid, lasting 2 to 18 months from mild global slowness of movement and gait until severe Parkinsonism [8,9,11,12]. Compared to cirrhotic patients without Parkinsonism, patients with Parkinsonism tend to be older, with higher MELD scores, higher incidence of hepatic encephalopathy episodes, and higher portosystemic shunts [13]. 

Parkinsonism in AHD is often accompanied by high levels of manganese in the blood, cerebrospinal fluid, and bilateral basal ganglia hyperintensities in T1WI MRI scans, reflecting cerebral manganese depositions [8,9,11,12,14]. The MRI findings reveal increased signal intensities on T2-weighted images involving the lenticular nuclei and the cerebellum’s brachium pontis or dentate nucleus. Other findings include increased signal on T1-weighted images in the basal ganglia, pituitary gland, quadrigeminal plate, caudate nucleus, subthalamic region, and red nucleus, probably reflecting intra-cerebral deposits of manganese [15,16]. High blood manganese levels and similar MRI abnormalities were demonstrated in cirrhotic patients without neurological manifestations [9,15,16,17].

The mortality rate in patients with Parkinsonism-related cirrhosis is high, reaching 54% in patients waiting for a transplant. Mortality rates remain high even after liver transplantation [13]. Despite the worse prognosis, there is a relief in the Parkinsonism symptoms and resolution of MRI findings after liver transplantation. This finding supports the strong association between cirrhosis and Parkinsonism [8].

2. Patients with cirrhosis who also suffer from PD

PD in cirrhosis may be misclassified as AHD based on MRI findings. At the same time, a minority of the allegedly AHD patients may be related to idiopathic PD based on clinical presentation, nuclear neuroimaging scans, and response to medical therapy [8,11,18]. The association between idiopathic PD and cirrhosis, including its co-prevalence and mutual influences on each disease trajectory, is not well-established. Mixed patterns of Parkinsonism in cirrhosis may represent different degrees of overlay or superimposition of idiopathic PD and AHD [9,11,19,20].

Occasionally, the neurological examination of patients with advanced liver disease reveals characteristics of idiopathic PD (i.e., slowly progressive asymmetrical resting tremor with late-onset postural and gait disturbances). It was previously stated that MRI pallidal hyperintensities in patients with cirrhosis and Parkinsonism are sufficient criteria for diagnosing AHD once other manganese exposure causes have been excluded [21]. Accordingly, most studies exploring the nature of AHD have considered cirrhotic patients with these MRI findings and clinical presentation suggesting idiopathic PD as a part of the AHD spectrum. The option of concomitant PD and cirrhosis with incidental basal ganglia hyperintensities was overlooked in many studies.

In various case series, a minority of the cirrhotic patients who exhibited extrapyramidal signs showed an idiopathic PD pattern. In nine cases of AHD, at least one showed predominant asymmetric resting tremors and a favorable response to levodopa, implying idiopathic PD [13]. No MRI results were presented. In a study of 11 cirrhotic patients with Parkinsonian AHD and MRI pallidal hyperintensities, one patient had asymmetric resting tremors without postural instability [4]. A trial of levodopa was not performed. In a series of eight patients with AHD, two subjects exhibited asymmetric resting tremors; one showed no improvement under levodopa, cabergoline, or post-liver transplantation, and the second died of hepatocellular carcinoma [6]. In a study of eight patients with Parkinsonian AHD and classical MRI abnormalities, two patients with primary biliary cirrhosis had asymmetric resting tremors and a positive response to levodopa [12]. 

A few studies highlighted idiopathic PD in cirrhosis, usually in absent T1WI basal ganglia hyperintensities [22]. Overall, the reviewed case series identified a variety of Parkinsonian AHD clinical subtypes without attempting to differentiate idiopathic PD in cirrhosis from AHD.

One promising way to differentiate idiopathic PD from AHD, complementary to the aforementioned clinical assessment, uses nuclear neuroimaging modalities. ^18^F-Dopa and dopamine transporter (DAT) positron emission tomography (PET) or single-photon emission computed tomography (SPECT) scans may be considered to evaluate the synaptic dopamine metabolism [11,23,24]. They can potentially identify classical PD molecular patterns and differentiate them from other patterns associated with AHD in ambiguous clinical cases (e.g., patients with predominant asymmetric resting tremor and T1WI pallidal hyperintensities) and may predict response to levodopa [24,25]. However, these tests are not commonly executed in clinical practice. They have not yet been validated for this purpose. Moreover, it is still unclear whether Parkinsonism in cirrhosis with classical PD molecular and clinical properties represents an incidental co-occurrence or a part of the AHD spectrum [23].

Several potential mechanisms may explain the described association. miR-129-5p significantly controls various pathways, genes, and proteins, impacting different diseases. It includes the WNT and PI3K/AKT/mTOR pathways, which, when dysregulated, lead to neurodegenerative diseases [26].

Based on a review of cross-sectional studies, fecal microbiota transplants could be an effective treatment for chronic liver diseases. This innovative therapy aims to deal with the microbial imbalances often found in cirrhotic patients and may also affect their neurological dysfunctions [27].

### 4.2. A Suggested Diagnosis Plan for Patients with Cirrhosis and Parkinsonism

As described above, there are several associations between Parkinsonism and cirrhosis. Discrimination between patients with simultaneous idiopathic PD and cirrhosis from patients with complex neuro-hepatic interactions resulting in AHD may have a prognostic and therapeutic significance.

Differentiation between AHD and other neurodegenerative disorders in patients with cirrhosis is essential for inequity prevention when registering patients for liver transplantation, as it may reverse AHD, and conservative pharmacological treatment is limited [23,28,29,30]. It may, to some extent, explain the variability in response to medical treatment or liver transplantation and can also help predict response to medical treatment [24]. Characterizing the specific pathological mechanisms involved in AHD may direct the development of targeted therapies [14]. Indiscrimination of these entities may result in false inference regarding the characterization of AHD. Thus, better diagnosis of subjects in the heterogeneous AHD group is necessary to improve understanding of AHD mechanisms, natural history, and treatment.

**Figure 1:**
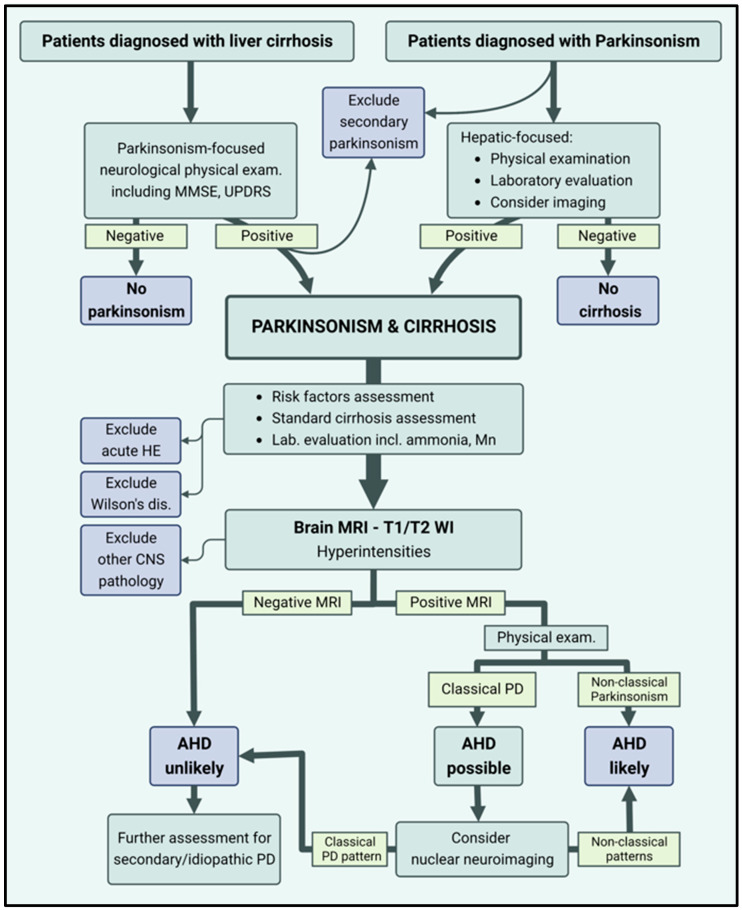
A suggested diagnostic plan for acquired hepatocerebral degeneration (AHD). MMSE: mini-mental state examination; UPDRS: unified Parkinson’s disease rating scale; HE: hepatic encephalopathy; CNS: the central nervous system; PD: Parkinson’s disease.

A suggested scheme for diagnosing patients who have cirrhosis and Parkinsonism is illustrated in Figure 1.

In patients with AHD, the diagnosis of liver cirrhosis usually precedes the diagnosis of Parkinsonism. However, the reverse temporal association has also been described [28,31,32,33]. Given the high prevalence of movement disorders among patients with cirrhosis, up to 60% in recent studies [7,8,34], we advocate a routine screening by a movement disorder specialist for every patient with cirrhosis, including a mini-mental state examination (MMSE) and unified Parkinson’s disease rating scale (UPDRS). We also recommend performing a focused physical examination for cirrhosis or portal hypertension stigmata and an essential laboratory evaluation of complete blood count, liver enzymes, and synthetic liver functions for all patients diagnosed with Parkinsonism. In selected cases with Parkinsonism, in which cirrhosis is suspected, liver imaging (e.g., abdominal ultrasound, elastography, computed tomography, or MRI) should be performed, followed by histopathologic examination and evaluation for the cause and complications of liver cirrhosis, according to the professional associations’ guidelines. Particular attention should be paid to the exclusion of Wilson’s disease in the setting of concomitant hepatic and neurological manifestations [11].

For all patients diagnosed with co-existing liver cirrhosis and Parkinsonism, a risk factors assessment [13] (e.g., the existence of portosystemic shunts, history of hepatic encephalopathy, or alcohol consumption) and a laboratory evaluation including blood manganese and ammonia levels should be performed, although of a limited value in the diagnosis and assessment of AHD [16]. Treatment with lactulose or Rifaximin should be considered individually to eliminate the contribution of acute hepatic encephalopathy to neurological manifestations [28].

Furthermore, we support brain MRI scans in all patients with Parkinsonism and cirrhosis, as T1WI basal ganglia hyperintensities are highly sensitive (but not specific) for AHD diagnosis [8,9,15,18]. In patients who do not exhibit these MRI abnormalities, AHD can be essentially ruled out. Then, an idiopathic PD or secondary Parkinsonism evaluation should be carried out. Neuroimaging is essential to exclude other causes of neurological manifestations such as chronic subdural hemorrhage, mass effect, or chronic microvascular ischemia. In all patients exhibiting Parkinsonian signs, secondary Parkinsonism due to medications (e.g., neuroleptics, antivirals, and immunomodulation), toxins (e.g., chronic exogenous manganese exposure in miners and metal workers, excessive seafood ingestion, or using manganese-rich total parenteral nutrition), genetic mutations (e.g., mutations in the SLC30A10 transporter which can cause familial manganese accumulation-induced Parkinsonism and liver injury), or vascular etiology should be excluded [11,14,28]. As aforementioned, the final diagnosis in ambiguous cases of Parkinsonism in cirrhosis can be supported by nuclear neuroimaging studies (PET or SPECT), but their utility for this purpose is still in its infancy [24].

The study has several limitations as it is a small retrospective cohort. More extensive prospective studies are necessary to support the described observations further.

## 5. Conclusions

In summary, our study, though modest in scale and qualitative, lends additional support to the hypothesis of chronic liver cirrhosis contributing to the development of Parkinsonism. Moreover, the proposed diagnostic scheme provides a potentially valuable tool for clinicians to identify and manage individuals at risk of developing Parkinson’s disease in the context of chronic liver cirrhosis. It may hold promise for therapeutic and prognostic applications. Further research is needed to investigate the underlying mechanisms responsible for these divergent clinical presentations and to depict the nuclear imaging characteristics of PD and AHD to provide a platform for guiding the treatment of these patients. 

## Data Availability

Data are available upon request.

## Abbreviations

PD: Parkinson’s disease; HCV: hepatitis C virus; HBV: hepatitis B virus; NASH: nonalcoholic steatohepatitis; AIH: autoimmune hepatitis; PBC: primary biliary cirrhosis; CNS: central nervous system; AHD: hepatolenticular degeneration; MELD: model for end-stage liver disease; PET: positron emission tomography; SPECT: single-photon emission computed tomography; MMSE: mini-mental state examination; UPDRS: unified Parkinson’s disease rating scale; HE: hepatic encephalopathy.
